# Supplementary material for: The MYB Transcription Factor GmMYB78 Negatively Regulates Phytophthora sojae Resistance in Soybean
Source: Int J Mol Sci. 2024 Apr 11;25(8):4247. doi: 10.3390/ijms25084247 (PMC11050205; doi:10.3390/ijms25084247)
Supplement: Supplementary file 1 [file ijms-25-04247-s001.zip › ijms-2939364-supplementary.pdf]

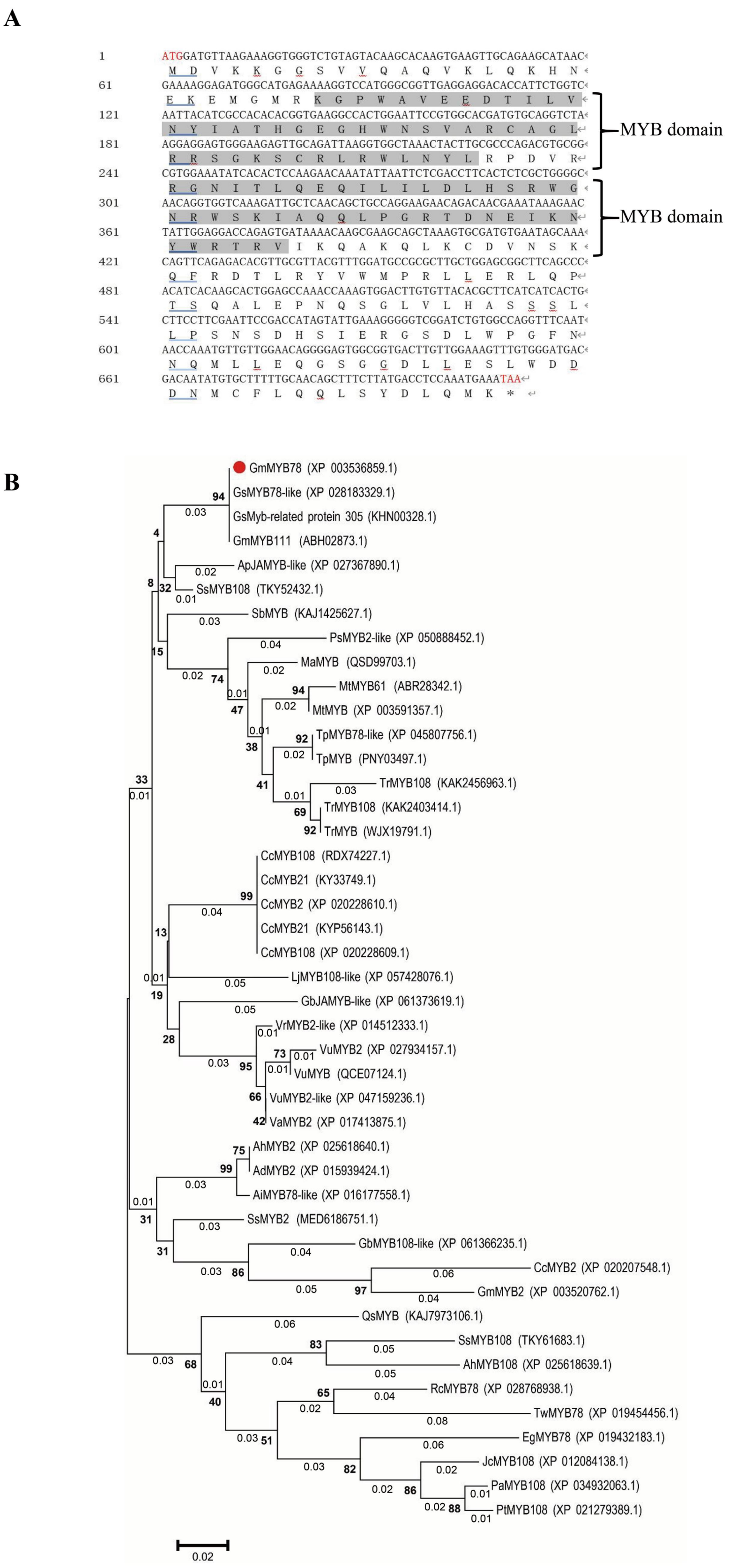

Figure S1. Nucleotide and amino acid sequences analyses of GmMYB78. (**A**)The MYB domain is shown in shadow. (**B**) Phylogenetic analysis of GmMYB78 with homologous genes.

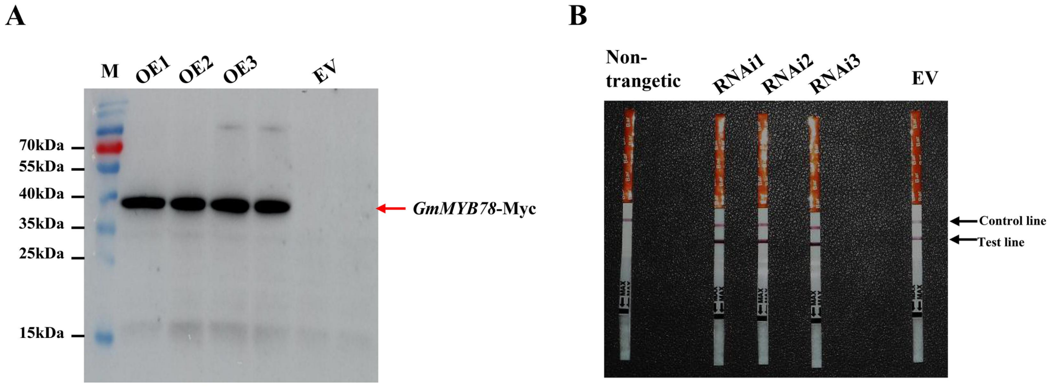

Figure S2. Analyses of *GmMYB78* transgenic soybean hairy roots. (**A**) Immunoblots showing the expression of the GmMYB78-Myc fusion protein in *GmMYB78*-OE transgenic hairy roots and the EV controls. The total protein extracts were analyzed using a 12% SDS-PAGE, and the immunoblot was probed with anti-Myc antibody. (**B**) The *GmMYB78*-RNAi and EV transgenic hairy roots were tested using QuickStix Kit for LibertyLink (bar) strips.

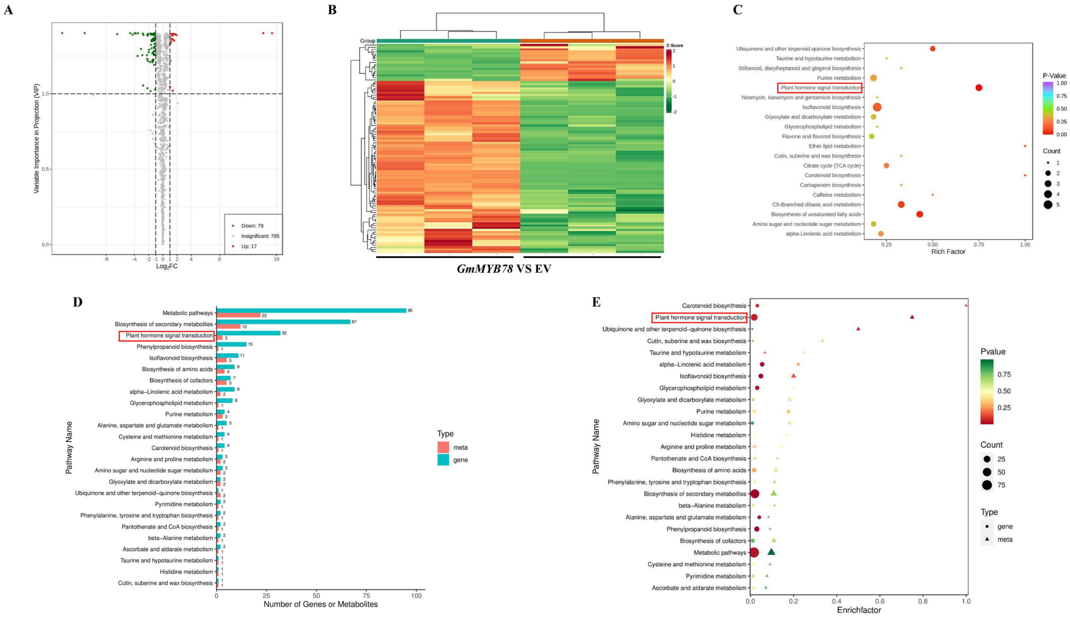

Figure S3. Metabolome analysis of metabolites in *GmMYB78* overexpression transgenic soybean hairy roots. (**A**) Volcano plots of significantly differentially metabolites in *GmMYB78*-OE vs. EV transgenic soybean hairy roots. (**B**) Heat map of significantly differentially metabolites between the EV and *GmMYB78*-OE transgenic soybean hairy roots. Using VIP ≥ 1 and fold-change set at ≥2 as the screening criteria, a total of 96 differentially metabolites were identified. (**C**) Metabolite annotation of KEGG classification of differentially metabolites. The differentially expressed metabolites are mainly involved in multiple biological processes, including plant hormone signal transduction. (**D**) KEGG classification bar chart of combined analysis of transcriptome and metabolome. The bar chart shows the number of differential metabolites and differential genes enriched to a certain pathway. (**E**) KEGG classification bubble chart of combined analysis of transcriptome and metabolome. Showing the top 25 pathways for P-value.

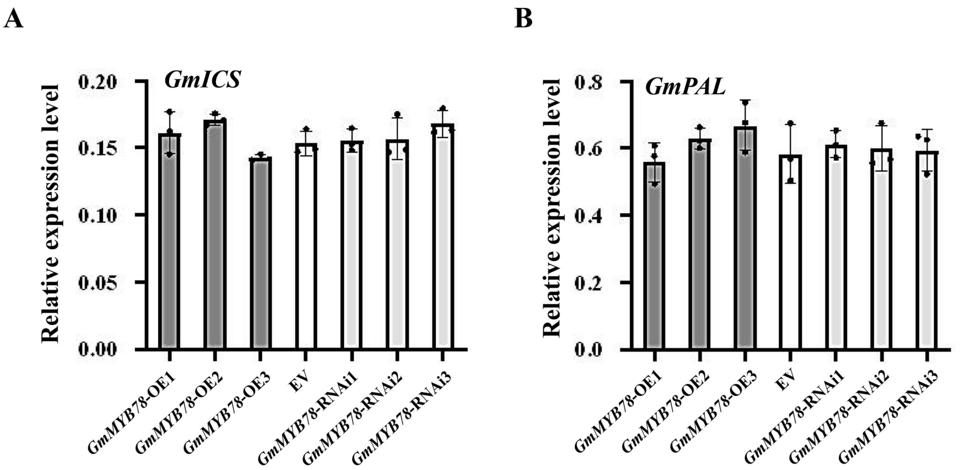

Figure S4. Investigation of the relationship between *GmMYB78* and the salicylic acid (SA) pathway in soybean. (**A,B**) Relative transcript level of *GmICS* (A) or *GmPAL* (B) in *GmMYB78*-OE, *GmMYB78*-RNAi and EV transgenic soybean hairy roots. The level of the control sample (EV lines) was set to unity. The reference gene *GmEF1β* and *GmTUB4* were used as reference genes. The experiment was performed on three biological replicates, each with three technical replicates, and was statistically analyzed using Student's *t*-test (*$P < 0.05$, **$P < 0.01$). Bars indicate the standard error of the mean.

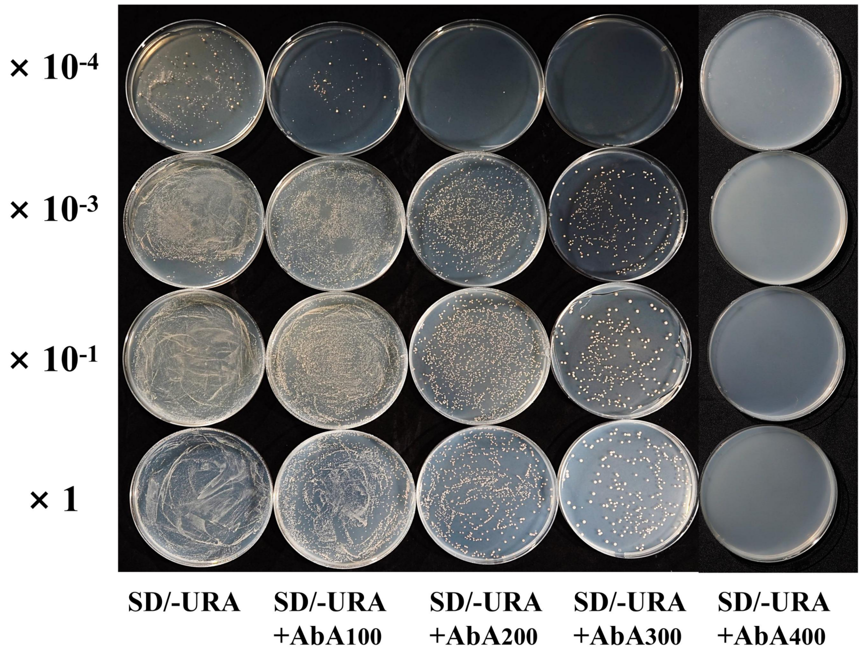

Figure S5. Screening of AbA concentration of Y1H [pBait-AbAi-*pGmbZIP25*] strain. ×1 represents the OD_{600} of the bait strain Y1H[pBait-AbAi-*pGmMYB78*] as 0.02; 10^{-1}, 10^{-2} or 10^{-3} represent dilutions of 10

times, 100 times, 1000 times, respectively. They were spread on solid SD/-Ura (SD medium without Ura) medium with different concentrations of AbA (100 ng/mL, 200 ng/mL, 300 ng/mL, 400 ng/mL) and incubated inverted on plates for 5 days.

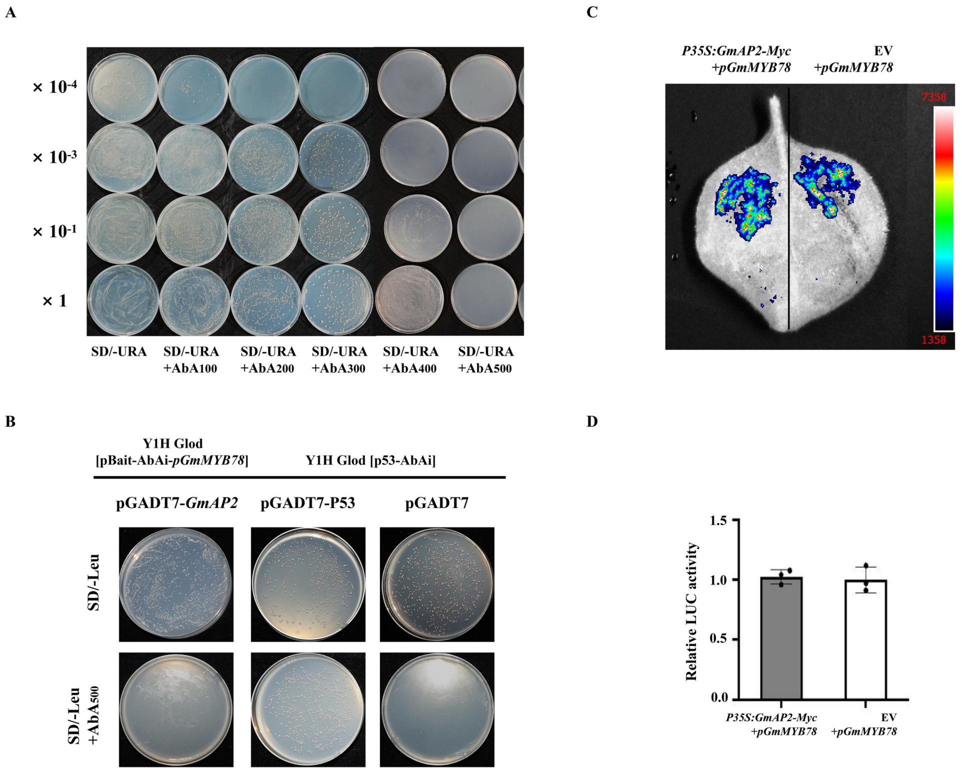

Figure S6. GmAP2 cannot directly regulate *GmMYB78*. (**A**) Screening of AbA concentration of Y1H [pBait-AbAi-*pGmMYB78*] strain. ×1 represents the OD_{600} of the bait strain Y1H[pBait-AbAi-*pGmMYB78*] as 0.02; 10^{-1}, 10^{-2} or 10^{-3} represent dilutions of 10 times, 100 times, 1000 times, respectively. They were spread on solid SD/-Ura (SD medium without Ura) medium with different concentrations of AbA (100 ng/mL, 200 ng/mL, 300 ng/mL, 400 ng/mL, 500 ng/mL) and incubated inverted on plates for 5 days. (**B**) Verification of GmAP2 regulate *GmMYB78* in yeast. (**C**) Dual-luciferase assay in *N. benthamiana* leaves showing that GmAP2 promotes the expression of *GmMYB78*. Representative photographs are shown. (**D**) Detection of LUC/Rluc activity to verify whether GmAP2 promotes the expression of *GmMYB78*. The combination of the reporter construct (*pGmMYB78*: LUC) and EV was used as a control. The experiment was performed on three biological replicates, each with three technical replicates, and the results were statistically analyzed using Student's *t*-test (*$P < 0.05$, **$P < 0.01$). Bars indicate the standard deviation of the mean (n=3).

Table S1. List of upstream protein of GmMYB78.

Library screening results by yeast one-hybrid

GenBank ID	Gene	Number of clones
XM-003519791.5	Zine-finger homeodomain protein	17
XM-014770182.3	Peroxisomal biogenesis factor 11family protein	1
XM-003522700.5	AT-hook motif nuclear-localized protein 17	2
XM-003543501.5	Transcription factor GmbHLH122	1

Table S2. List of primers used in this study.

Primers for quantitative real-time PCR

Primer name	Primers (5′-3′)
GmMYB78-RT-qPCR	F: GGAGGAGTGGGAAGAGTTGC
	R: GTGATATTTCCACGCCGCAC
GmTUB4- RT-qPCR	F: GGCGTCCACATTCATTGGA
	R: CCGGTGTACCAATGCAAGAA
GmEF1β- RT-qPCR	F: CCACTGCTGAAGAAGATGATGATG
	R: AAGGACAGAAGACTTGCCACTC
TEF1- RT-qPCR	F: TGATCGTGCTGAACCACCC
	R: CGAGCGACGGTCCATCTT
PSPEL1- RT- qPCR	F: CCGCGT ACG TGGC TT TGGTGAG
	R: ATCT TGGCGAC TGAGGC TGC T TAC
GmPR1- RT-qPCR	F: GCCCTTATCGGGGTGTTTCT
	R: TCCTTTCTTCCTGTGTGTATTGC
GmPR2- RT-qPCR	F: CTTCCGTATGTGTTGCTTCGT
	R: TTTCCTGTATTGTGCCGCCTT
GmPR3- RT-qPCR	F: TTCTCCTTCTCCTCTTCTCCTT
	R: TGCCTTTGCCTACTGTATGT
GmPR10- RT-qPCR	F: TTCGCCGTGTGTGCTTATTGT
	R: TATCCTCCTCTTCCTCTATTGTC
GmAOS1- RT-qPCR	F: AAGTGCATCACTGACGAGGG
	R: TGTGGGAACCCACCTTCAAC
GmJAZ1- RT-qPCR	F: AGTGGTGTGGCTCAGGAATG
	R: GAGGGGCAAGGGCTATGTA
GmJAZ2- RT-qPCR	F: GCAGAAGAACAACAACGACGG
	R: CGAGGGGGTTTGACGTAGAG
GmbHLH122- RT-qPCR	F: TGCCACTCACCCAAGAA
	R: CCACAGCCAAGTCCAAC

Primers for gene cloning

Primer name	Primers (5′-3′)
GmMYB78	F: ATGGATGTTAAGAAAGGTGGGT
	R: TTATTTCATTTGGAGGTCATAAGA
GmbHLH122	F: ATGGAGTCAGATCTCGAGCAAC
	R: TTATTGTTGCTTCTCGTGTGA

Primers for constructs in transformation of soybean hairy roots

Primer name	Primers (5′-3′)
GmMYB78-Myc	F:CGGGGGACTCTTGACCATGGAAATGGATGTTAAGAAAGGTGGGT

	R: CCCACGTGGCTAGC AGATCT TTTCATTTGGAGGTCATAA
GmMYB78 RNAi1	F:TTTCATTTGGAGAGGACACGCTCGAGATGGATGTTAAGAAAGGTGGGTC<br>R:AATCATCGATTGGGCGCGCCCCATGGTTCGCTTGTTTTATCACTCTGGT
GmMYB78 RNAi2	F:TCCCGGGTCTTAATTAACTCTCTAGAATGGATGTTAAGAAAGGTGGGTC<br>R:GTCAATTTGCAGGTATTTGGATCCTTCGCTTGTTTTATCACTCTGGT

Primers for constructs in yeast one-hybrid assays

Primer name	Primers (5′-3′)
pGmMYB78	F: TTAACATATTAGATTGTGTAAAA<br>R: ACTTATTGGGGGCATGTGGAGTT
pGmMYB78-pBait-AbAi	F: AAAAGCTTGAATTCGAGCTCTTAACATATTAGATTGTGT<br>R: TACAGAGCACATGCCTCGAGACTTATTGGGGGCATGTG
GmbHLH122-AD	F: CCATGGAGGCCAGTGAATTCATGGAGTCAGATCTCG<br>R: AGCTCGAGCTCGATGGATCCTTATTGTTGCTTCTCGT
pGmbZIP25	F: CTATTTGATCCATACACATA<br>R: AGAAGGAGGAAGGAAAGAG
pGmbZIP25-pBait-AbAi	F: AAAAGCTTGAATTCGAGCTC CTGTTGTCTATCAACGGATAAA<br>R:TACAGAGCACATGCCTCGAGAAGCTGATTCTCTGTTGCTGTTG

Primers for subcellular localization

Primer name	Primers (5′-3′)
GmMYB78-1302	F: CGGGGGACTCTTGACCATGGATGTTAAGAAAGGTGGGT<br>R: AGATCTGACTAGT TTATTTCATTTGGAGGTCATAAGA

Primers for transient expression assay

Primer name	Primers (5′-3′)
GmMYB78-BD	F: TGGCCATGGAGGCCGAATTCATGGATGTTAAGAAAGGTGG<br>R: CGCTGCAGGTCGACGGATCCTTATTTCATTTGGAGGTCATAA

Primers for transient transcription dual-luciferase assay

Primer name	Primers (5′-3′)
pGmMYB78-LUC	F: GTCGACGGTATCGATAAGCTTTTAACATATTAGATTGTGT<br>R: CGCTCTAGAACTAGTGGATCCACTTATTGGGGGCATGTG
GmbHLH122-3301	F: CGGGGGACTCTTGA CCATGGAAATGGAGTCAGATCTCG
pGmbZIP25-LUC	R: CCCACGTGGCTAGC AGATCTTTGTTGCTTCTCGT<br>F: GTCGACGGTATCGATAAGCTTCTATTTGATCCATACACATA<br>R: CGCTCTAGAACTAGTGGATCCAGAAGGAGGAAGGAAAGAG

Primers for transcriptome sequencing analysis

Primer name	Primers (5′-3′)
GmPRR13 -qPCR	F: AAGTGCATCACTGACGAGGG<br>R: TGTGGGAACCCACCTTCAAC

GmbZIP25-qPCR	F: AGTGGTGTGGCTCAGGAATG
	R: GAGGGGCAAGGGCTATGTA
GmMYB177-qPCR	F: GATGCTTCCCTTTTGGCTCCTT
	R: AACAACGGAGCCGTGCAAA
GmPR-4A -qPCR	F: ACCCTGAGCAGATTGGTTGG
	R: ATCCTCACCGTAGCCTCAGT
GmLOX9-qPCR	F: GTCCATCAGTATTGGTGTAG
	R: GTCCATCAGTCTTGGTGGCA
GmCHS13-qPCR	F: GAAGCTGGGAAAACAAGCCG
	R: GAGAAGCTTCGTGAGCTGGT
GmBVT -qPCR	F: TGGCCCCGGAACCATTAAA
	R: CTCCACTGTGTCTGGCAACC
GmARM -qPCR	F: ACTAGCACTGGTGGTGTTGG
	R: TGCCGTCATCCAAGTTTCCA
